# Quality of emergency obstetric and newborn care services in Wolaita Zone, Southern Ethiopia

**DOI:** 10.1186/s12884-022-05019-w

**Published:** 2022-09-06

**Authors:** Mihiretu Alemayehu, Bereket Yakob, Nelisiwe Khuzwayo

**Affiliations:** 1 School of Public Health, College of Health Sciences and Medicine, Wolaita Sodo University, Wolaita Sodo, Ethiopia; 2 School of Nursing and Public Health, Discipline of Public Health, University of KwaZulu-Natal, Durban, South Africa; 3 School of Population and Public Health, the University of British Columbia, Vancouver, BC Canada

**Keywords:** Healthcare quality, Emergency obstetric care, Newborn care, Emergency obstetric and newborn care, Observed quality, Health system, Maternal health

## Abstract

**Background:**

Globally, nearly 295,000 women die every year during and following pregnancy and childbirth. Emergency obstetric and newborn care (EmONC) can avert 75% of maternal mortality if all mothers get quality healthcare. Improving maternal health needs identification and addressing of barriers that limit access to quality maternal health services. Hence, this study aimed to assess the quality of EmONC service and its predictors in Wolaita Zone, southern Ethiopia.

**Methodology:**

A facility-based cross-sectional study was conducted in 14 health facilities. A facility audit was conducted on 14 health facilities, and 423 women were randomly selected to participate in observation of care and exit interview. The Open Data Kit (ODK) platform and Stata version 17 were used for data entry and analysis, respectively. Frequencies and summary statistics were used to describe the study population. Simple and multiple linear regressions were done to identify candidate and predictor variables of service quality. Coefficients with 95% confidence intervals were used to declare the significance and strength of association. Input, process, and output quality indices were created by calculating the means of standard items available or actions performed by each category and were used to describe the quality of EmONC.

**Result:**

The mean input, process, and output EmONC services qualities were 74.2, 69.4, and 79.6%, respectively. Of the study participants, 59.2% received below 75% of the standard clinical actions (observed quality) of EmONC services. Women’s educational status (B = 5.35, 95% C.I: 0.56, 10.14), and (B = 8.38, 95% C.I: 2.92, 13.85), age (B = 3.86, 95% C.I: 0.39, 7.33), duration of stay at the facility (B = 3.58, 95% C.I: 2.66, 4.9), number of patients in the delivery room (B = − 4.14, 95% C.I: − 6.14, − 2.13), and care provider’s experience (B = 1.26, 95% C.I: 0.83, 1.69) were independent predictors of observed service quality.

**Conclusion:**

The EmONC services quality was suboptimal in Wolaita Zone. Every three-in-five women received less than three-fourths of the standard clinical actions. The health system, care providers, and other stakeholders should emphasize improving the quality of care by availing medical infrastructure, adhering to standard procedures, enhancing human resources for health, and providing standard care regardless of women’s characteristics.

## Introduction

Globally, approximately 810 women and 6500 newborns die every day from preventable causes related to pregnancy and childbirth. Sub-Saharan Africa and South Asia account for 86% of global maternal mortality [1, 2]. In 2020, an estimated 2.4 million neonates died, with about a third of all neonatal deaths occurring within the first day after birth and close to three quarters occurring within the first week of life [2]. The high maternal and neonatal deaths in low-income countries and different socioeconomic statuses reflect inequalities in access to quality health services. There is a stark inequity in maternal mortality ratio (MMR), i.e., 462 per 100,000 live births in low-income countries versus 11 per 100,000 live births in high-income countries [3].

The major causes of maternal deaths are known and preventable. Women die due to complications during and following pregnancy and childbirth [3]. The main complications that account for nearly three-fourths of all maternal deaths are hemorrhage, infections, hypertensive disorders of pregnancy, obstructed labor, and unsafe abortion. The World Health Organization (WHO) reported that the three leading causes (prematurity, intrapartum-related complications, and sepsis) account for the majority of neonatal death in 2022 [4]. Access to high-quality care during pregnancy and intrapartum time can significantly reduce maternal and neonatal mortality rates [5].

The World Health Organization identified Emergency Obstetric and Newborn Care (EmONC), the care given to mothers and neonates during pregnancy, child delivery, and postpartum when facing severe and life-threatening complications, as an essential intervention in preventing maternal deaths. It can avert nearly 75% of maternal deaths if all mothers get quality health care [6]. Evidence shows that it is not mere contact (access) with the health facility or care provider that results in better health outcomes, but the actual content of care and process of delivery that can reduce morbidity and mortality [7].

Evidence shows that the health service delivered is inadequate and of poor quality, especially in low and middle-income countries (LMICs)**.** Recently, poor service quality has been a more significant challenge for reducing mortality than insufficient access. A quality service provision can save over eight million lives each year in LMICs [8]. Several studies underlined the necessity of quality obstetric care to curb the high maternal morbidity and mortality levels in developing countries [7, 9, 10].

Enhancing the quality of health care service is one of the priority health strategic directions in Ethiopia. Though the Ethiopian government aimed to reduce the maternal mortality ratio and neonatal mortality rate [11], none of the targets were achieved, although encouraging progress has been made [12]. For instance, the Ethiopian Health Sector Plan II (HSTP 2020/21–2024/25) and Ethiopian National Health Care Quality Strategy emphasized the provision of quality obstetric services and the reduction of maternal and neonatal mortality [11, 13].

However, Ethiopia remains one of the countries with the largest MMR globally, i.e., 401 maternal deaths/100,000 live births, nearly twice as high as the world average of 211 maternal deaths/100,000 live births in 2017 [14]. Besides, according to the Ethiopian EmONC assessment report in 2016, 66% of mothers gave birth at health institutions. Regardless of complications, every childbirth should occur in a health facility that can readily manage/treat obstetric emergencies. However, only 14% of them gave birth in EmONC facilities, indicating that most facilities were not ready to treat obstetric emergencies adequately. This has shown a shortage of EmONC facilities to treat obstetric emergencies [15]. Besides, to achieve the global SDG goals, the Ethiopian HSTP II targeted to reduce maternal mortality from 401 per 100,000 live births to 279 and neonatal mortality from 33 per 1000 live births to 21 in 5 years [11], necessitating the need for interventions that target on the major causes of death.

Therefore, providing evidence for timely intervention in maternal health is one of the WHO’s priority plans. Hence, improving maternal health needs identification and addressing barriers limiting access to quality maternal health services [3]. One of the most commonly used frameworks for health service quality assessment is Donabedian’s framework. This framework uses the triad of *structure*, *process*, and *outcome* of health care. It defined *structure* as the settings, qualifications of providers, and administrative systems through which care takes place; *process* as the components of care delivered; and *outcome* as recovery, restoration of function, and survival. These concepts, therefore, remain the foundation of quality assessment today [16, 17].

Despite the significant advancement of Donabedian’s framework for a health performance metric, much of the research up to now has been focusing on only a part of the triads, had a different objective, or used a different measurement approach [18–23]. Although some studies investigated the quality of EmONC services in Sub-Saharan African countries [23, 24], using valid indicators of quality of care in resource-poor countries and providing a comprehensive report on the structure, process, and outcome components of care lacked [25]. Some studies identified the predictors of delivering poor quality of care such as lack of medical communication, proper standards and guidelines, policies, and specific action plans [21, 23, 26]. Nevertheless, the findings’ objectives, study settings, and generalizability implied the need for evidence on the predictors of quality EmONC service provision. So far, no previous study has investigated the quality of EmONC services and predictors in Ethiopia.

Saving the lives of women and neonates (as maternal and newborn health are closely linked) needs high-quality care in pregnancy and during and after childbirth [3]. However, evidence shows that ensuring service quality at facilities remains a challenge for Ethiopia [27]. Hence, this study aimed to assess the quality of EmONC services and predictors in Wolaita zone, southern Ethiopia, using Donabedian’s quality assessment framework [16], emphasizing the observed quality of EmONC services.

## Methods

### Study area and design

The study was conducted in Wolaita Zone, southern Ethiopia, 330 km southwest of Addis Ababa, the capital of Ethiopia. The Zone’s population was projected to be more than 2.6 million in 2020 [28, 29]. The 2020 Wolaita Zone Health Department report indicated ten hospitals (one referral hospital, two general hospitals, seven primary hospitals), 70 health centres, and 326 health posts (Wolaita Zone Health Department: Annual Progress Report, Unpublished). The facilities provide preventive, curative, and rehabilitative health services for over 2 million people in the Zone and neighboring zones [28]. Accordingly, the Zone had 80 EmONC facilities. Of them, two non-governmental and eight government hospitals provided Comprehensive Emergency Obstetric and Newborn Care (CEmONC) services, and 70 health centres provided Basic Emergency Obstetric and Newborn Care (BEmONC) services in 2019 (Wolaita Zone Health Department: Annual Progress Report, Unpublished).

A facility-based cross-sectional study was conducted to assess the quality of EmONC service and its predictors in Wolaita Zone, southern Ethiopia. The study was conducted from October 01 – December 31, 2020.

### Population

The source population are all EmONC facilities and women and newborns who came for EmONC services in Wolaita Zone during the study period. Randomly selected health facilities and women and their newborns were the study populations of the study.

### Eligibility criteria

Women aged 18 years and above who visited EmONC facilities during the study period were included in the study. In contrast, women referred to another health facility and those who had major obstetric/gynecological surgical procedures (cesarean section, hysterectomy, colporrhaphy, cervical cerclage, etc.) were excluded from the study.

### Sampling and sample size

#### Sampling of health facilities

Of the 22 districts of Wolaita Zone, seven (30% of the total) districts were randomly selected. There were 27 eligible health facilities in the randomly selected districts. To take a representative sample from the selected districts, we included 14 facilities (more than 50%) from the total eligible health facilities in the districts. Accordingly, two EmONC facilities from each district were randomly selected, making the total number of health facilities selected for the study 14.

#### Sampling of women

The sample size for observation of EmONC services and exit interview was calculated using the *single population proportion formula* based on the following assumptions: a-47% proportion (p) of women who received quality EmONC services in Tanzania [30], the normal distribution of z at 95% confidence interval, and 5% margin of error (d). To adjust for non-responses, the sample size was increased by 10%, making the required sample size for the study 422. This sample size was intended to assess the process and outcome components of EmONC service quality. The calculated sample was allocated to the facilities considering the previous year’s volume of EmONC services’ utilization. Accordingly, the proportional allocation ranged from 82 in Wolaita Sodo University comprehensive specialized hospital to 9 in Wadu health centre as per their volume of EmONC service utilization. The sample size calculation using the single population formula is shown below

$$n=\frac{z^2p\left(1-p\right)}{d^2}$$

$$\frac{1.96^2\ast 0.47\left(1-0.47\right)}{0.05^2}=383$$

After adding 10% non-response rate, the final sample size required for the study was 383 + 39 = 422.

On the other hand, all selected women who came for EmONC services were recorded. Using the K^th^ number generated, women were selected systematically as they came for EmONC services. In the previous year, 9211 women (2303 patients in 3 months) visited the selected facilities for obstetric emergencies, presenting grounds to calculate the k^th^ number to choose eligible women systematically from the facilities. Accordingly, every fifth woman was selected for the study until the required sample size was met in each facility.

### Data collection instrument

The triad of *structure, process, and outcome* of the Donabedian Framework for Health Care Quality [16], was used to assess the utilization of quality of EmONC services. The structural (input) quality of care was measured using a facility audit checklist that was also developed after reviewing the literature [15, 18–20, 31]. A structured EmONC services observation checklist was developed after reviewing different guidelines and instruments [16, 18, 19, 31] and was used to observe the EmONC processes, i.e., to measure the observed quality. The EmONC service delivery (process) observation and facility audit checklists were prepared and used in the English language. The exit interview tool (questionnaire) was developed by the investigators after reviewing the literature [15, 18–20, 31]. The exit interview tool contained items regarding the socio-demographic characteristics of women, factors associated with the quality of EmONC services, and output quality assessment items. The exit interview tool was developed in English, translated into the local language (Wolaita Dona), and re-translated into English to check the consistency.

### Data collection

A two-day training was given to the data collectors. The data were collected by 14 data collectors who had a BSc in nursing (midwifery) and had experience with collecting data with the Open Data Kit (ODK) application and had no history of working in the assigned health facility. Similarly, seven supervisors, who had MPH and experience with data collection and supervision were hired to collect the data. The data were collected using the ODK mobile application with android tablet phones. The data collectors filled the facility audit, EmONC care observation checklist, and exit interview questions loaded in the ODK. ODK submitted the data to an online server in real-time. One supervisor was assigned to two health facilities, checked the data collection processes, provided support for data collectors on-site, and provided feedback to them in real-time.

#### Facility audit data collection

The facility audit was conducted 1 week before the observation of EmONC services and exit interviews. Seven data collectors conducted the facility audit. They completed the different sections of the audit checklist by contacting the heads of the units of the health facilities such as the health facility manager/director, maternal and child health unit heads, pharmacy and laboratory unit heads, and document reviews. Additional staff was consulted for information that was not available by the above persons or on their referral.

#### Observation of EmONC services data collection

The data collectors enrolled the woman if she met the inclusion criteria and documented the care provided to the woman with the EmONC services observation checklist. The observation of EmONC care started at the initial patient assessment, followed by all the stages of labor, and ended up at discharge from the facility. This approach was supported by other studies [15, 18, 19, 31].

#### Exit interview data collection

The data collectors interviewed the woman after 6 hours of postpartum, or a discharge summary was issued to her; whichever came first was sufficient to initiate the interview. The exit interview was done privately in a room in the facility.

### Data management and quality control

Before the data collection, a pre-test was conducted in a similar setting (out of the study area) to check for the appropriateness of the study tools. Regular supervision was provided by the principal investigator, co-researchers, and supervisors to the data collectors to check for completeness, and confusion was cleared at the end of each data collection day.

Since the study involved observation of the care process by health workers, ruling out the Hawthorne effect was impossible. However, several considerations were made to minimize the effect of the presence of observers on the providers’ behavior. Initially, the data collectors assured the care providers that the purpose of the study was not for evaluating their performance or reporting it to their supervisors. Besides, observers had informed care providers that individual data will not be shared publicly (published reports only refer to aggregate data). The investigators discarded the first five observations of each health care provider because studies reported that care providers reverted to their normal behaviors after being observed a few times (observations) by the same observers [32–34]. In addition, care providers were not aware of the items on the checklist, so they could not prepare in any way. For further caution, the data collectors were not assigned to facilities where they currently or previously worked.

### Data analysis

The data were exported to Stata v17 (College Station, Texas) to clean, re-code, explore and do advanced analysis. The descriptive statistics were done using frequency tables, charts, and summary statistics. The principal component analysis (PCA) was conducted to determine the household wealth index of study participants using the DHS approach [12]. The simple and multiple linear regression analyses were done to identify candidate and predictor variables of the index (discussed below) of the observed quality of EmONC services. Coefficients with a 95% confidence interval were used to declare the significance and strength of association. Variables with a *p*-value less than 0.25 in the simple linear regression were taken as a candidate for multiple linear regression, and those with a *p*-value below 0.05 in the final model (multiple linear regression) were declared independent predictors of the observed quality of EmONC services.

Linear assumptions, such as homogeneity of variances and normality were checked and fulfilled. In the multi-collinearity test, all predictor variables had a variance inflation factor (VIF) value below 5. The final model was found significant with the adjusted *R*^2^ value of 0.344, explaining 34.4% of the variation.

### Ethical considerations

The study was conducted after receiving ethical approval from the University of KwaZulu-Natal Biomedical Research Ethics Committee (BREC) (Ref: BREC/00001744/2020), South Africa, and the Institutional Review Board (IRB) of the College of Health Sciences and Medicine Wolaita Sodo University (Ref: CARD 4/979/20), Ethiopia. Furthermore, permission to conduct the study was obtained from Wolaita Zone Health Department and all participating health facilities. Written informed consent was obtained from all participants. The participants were informed that they had full right to participate or not in the study. Furthermore, the objectives, benefits, and harms of research were communicated. Respondents were also informed that their responses would be kept confidential. During observation of care provision, the data collectors were passively observing (did not intervene) the EmONC care provided to women.

### Operational definitions

- **Emergency Obstetric and Newborn Care:** is the care given to mothers and neonates during pregnancy, child delivery, and the postpartum period when she faces serious and life-threatening obstetric complications [15, 35].
- **Obstetric complication:** a woman is classified as having obstetric complication if she had at least one of these; a) hemorrhage (antepartum and postpartum), b) prolonged and/ or obstructed labor, c) postpartum sepsis, d) complications of abortion, e) severe pre-eclampsia and eclampsia, f) ectopic pregnancy and g) ruptured uterus [15, 35].
- Input quality: was measured using an index created by calculating the mean of performance of 75 items (66 items of structure and nine items of signal function tests) and computed out of 100%. The index included items that assess power and water supply, waste management, drugs, supplies, equipment, storage, examination room, delivery room, waiting area, and standard precautions. It also includes the nine items (seven for BEmONC and nine for CEmONC facilities) of signal functions tests to assess the readiness of EmONC facilities in the past 3 months preceding the study [31, 35, 36].
- Observed quality: was measured using an index created by calculating the mean of performance of 42 items related to EmONC services (standard clinical actions) and computed out of 100%. The index includes items that assess the observed quality of care EmONC service, including general patient assessment and danger signs, standard precautions, standard procedures in the stages of labor, and communication [31].
- Output quality: was assessed by using the woman’s satisfaction with the EmONC services she utilized. A total of 12 items using a 5-scale Likert scale tool (ranging from “strongly disagree” to “strongly agree”) were used to assess the woman’s satisfaction with the EmONC services given at the health facility and computed out of 100% [37]***.***
- Basic Emergency Obstetric and Newborn Care (BEmONC) services: are expected to provide the seven signal function tests, namely: parenteral antibiotics, parenteral uterotonics, parenteral anticonvulsants, manual removal of placenta, removal of retained products, assisted vaginal delivery, and neonatal resuscitation [31].
- Comprehensive Emergency Obstetric and Newborn Care (CEmONC) services: are expected to provide the seven services given by BEmONC facilities and the additional two services, namely, caesarian section and blood transfusion services [31].

## Result

### Socio-demographic characteristics

A total of 414 women participated in the study in 14 health facilities making a 98% response rate. The mean age of the women was 28.2 years with a standard deviation (SD) of 5.4 years, and it ranged from 18 to 40 years. Nearly two-thirds of them were housewives and protestants, while almost all (98.8%) of them were married. Roughly half (229) of them used an ambulance, while 78 (18.8%) walked to the health facility. The mean family size of study participants was 4.4 (SD = 1.7) (Table 1).

**Table 1:** Socio-demographic characteristics of study participants, Wolaita Zone, southern Ethiopia (*n* = 414)

Variable	Category	Frequency	Percent
Age	< 25 years	102	24.6
	25–30 years	182	44.0
	Above 30 years	130	31.4
Education	Not attended at all	65	15.7
	Grade 1–8	142	34.3
	Grade 9–12	141	34.1
	College/university	66	15.9
Marital status	Married currently	409	98.8
	Unmarried currently	5	1.2
Religion	Protestant	289	69.8
	Orthodox	109	26.3
	Others ^a^	16	3.9
Occupation	Employed (Farmer)	250	60.4
	Employed (Other sectors)	59	14.3
	Student	65	15.7
	Others ^b^	40	9.7
Means of transportation used to arrive at the facility	Ambulance	229	55.3
	Walked on foot	78	18.8
	Other motor vehicle ^c^	107	25.8
Distance from the facility (in minutes)	< 30	303	73.2
	30 and above	111	26.8
Family size	< 5	249	60.1
	Five and above	165	39.9

^a^ Muslim, traditional, apostolic; ^b^ daily laborer, housewife, merchant; ^c^ car, motorbike, bajaj

### Obstetric conditions and outcomes

**Fig. 1:**
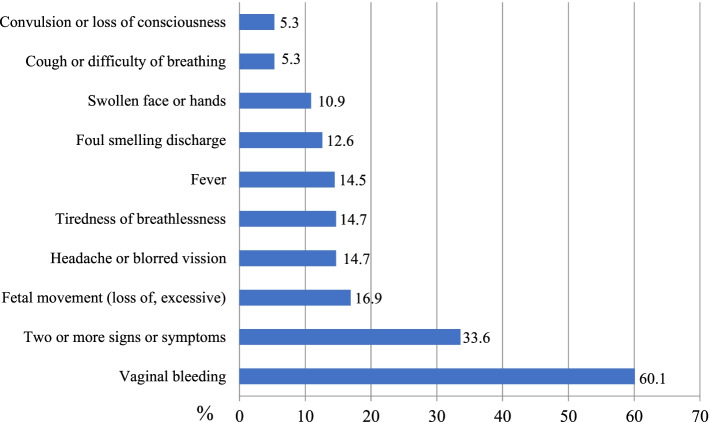
Signs and symptoms of obstetric complications when study participants arrived at the health facilities, Wolaita Zone, southern Ethiopia (*n* = 414)

EmONC services observation showed that 249 (60.1%) and 70 (16.9%) of the women visited the health facility for vaginal bleeding and issues related to fetal movement, respectively (Fig. 1). Most (91.3%) of the observed women gave birth to live-born babies, whereas 27 (6.5%) of the mothers had abortions, and 9 (2.2%) had stillbirths. Midwives attended Two-third (287) of mothers, and 103 (24.9%) were treated by care providers with more than 10 years of work experience. Of 414 women, 107 (25.8%) received EmONC services in a private room, while 307 (74.2%) women received the care in shared rooms that did not maintain their privacy (Table 2).

**Table 2:** Obstetric conditions and outcomes of the study participants, Wolaita Zone, southern Ethiopia (*n* = 414)

Variable	Category	Frequency	Percent
Duration of stay at the health facility	One day	337	81.4%
	More than one day	77	18.6%
Number of women in one delivery room	One patient	107	25.8%
	Two patients	171	41.3%
	Three and above patients	136	32.9%
Pregnancy outcome (current)	Live born baby	378	91.3
	Stillbirth	9	2.2
	Abortion	27	6.5
Mode of current delivery	SVD	362	87.4
	Assisted (instrumental) or abortion	52	12.6
Episiotomy was done (current delivery)	No	268	64.7
	Yes	146	35.3
Delivered previous child at any health facility	No	23	5.6
	Yes	391	94.4
ANC follow-up for this pregnancy	No	45	10.9
	Yes	369	89.1
Place of ANC follow-up (*n* = 369)	This health facility	233	63.1
	Another health facility	136	32.9
Patient’s sex preference of care provider	Male	180	43.5
	Female	234	56.5
Sex of care provider (*n* = 136)	Male	101	74.3
	Female	35	25.7
Women served by the care provider (qualification)	General practitioner	49	11.8%
	Specialist obstetrician/gynecologist	28	6.8%
	Nurse	20	4.8%
	Midwife	287	69.3%
	IESO	30	7.2%
Women served by the care provider (sex)	Male	169	40.8%
	Female	245	59.2%
Women served by the care provider (experience)	<=5 years	150	36.2%
	6–10 years	161	38.9%
	> 10 years	103	24.9%

### Input quality

The mean of the structural quality of EmONC services was 74.2% (95% C.I: 71.1, 77.1%, SD = 5.9%). The mean varied by facility, ranging from 63.3 to 85%. Eight of the 14 facilities fulfilled at least 75% of the input quality index. Of the 14 health facilities, three had a shortage of beds (for admission after delivery or abortion service is given), such that patients were obligated to share beds or sleep on the floor. All the assessed health facilities had an electric power supply, separate room for delivery, oral rehydration salt, cord ties/clips, and a baby weighing scale. Low-reading thermometers and solar refrigerators were available at two facilities. However, except one referral hospital, all audited facilities did not provide food for patients (Table 3).

**Table 3:** Input quality of EmONC service in Wolaita Zone, Southern Ethiopia (*n* = 14)

S. N	Item	Number of facilities
	**Power, water supply, and waste management**	
1.	Facility connected to the electric supply	14
2.	The facility has running water supplied to the labor and delivery care rooms	6
3.	The toilet is in functioning condition for general staff use	10
4.	The toilet is in functioning condition for patient use	12
5.	The toilet is in functioning condition in the labor ward for patient use	4
6.	Filled oxygen cylinder with cylinder carrier and key to open valve	10
7.	Liquid spills/trash on the floor is invisible by observation	11
	**Drugs, supplies, and equipment**	
8.	Blood pressure cuff	13
9.	Fetal stethoscope	12
10.	Kidney basins	13
11.	Sponge bowls	13
12.	Clinical thermometer	13
13.	Low reading thermometer (32 °C or 35 °C)	2
14.	Suture needles/suture materials	12
15.	Catheter for IV line/adult cannula (16–18)	14
16.	IV Infusion stand(s)	14
17.	Urinary catheters	14
18.	IV cannula 24 gauge	14
19.	Dipstick for urinalysis	12
20.	Adult ventilator bag and mask	10
21.	Wheelchair	10
22.	Stretcher	12
23.	Examination table	14
24.	Labor/delivery table	14
25.	Dressing forceps	13
26.	Partograph form	13
27.	Watch or clock that can be easily seen	5
28.	Measuring tape	14
29.	Obstetric wheel (for measuring gestational age)	8
30.	Water filter	4
31.	HIV rapid testing kit	13
32.	Have steroids	11
33.	Have antimalarials	12
34.	Have any antiretrovirals	12
35.	Have any contraceptives	14
36.	Have Vitamin K (for a newborn)	12
37.	Have Chlorhexidine (4% gel for cord cleansing)	10
38.	Have Nystatin (for a newborn)	5
39.	Have Oral rehydration solution	14
40.	The facility provides food for patients	1
	**Storage**	
41.	Drug inventory register/system	14
42.	At least one functioning electric/gas refrigerator other than an EPI refrigerator	13
43.	At least one functioning solar refrigerator other than an EPI refrigerator	2
	**Examination room, delivery room, and waiting area**	
44.	There are empty beds for the next patients	6
45.	Separate room/space for labor (first stage)	13
46.	Separate room/space for delivery	14
47.	Separate room/space for maternity room for complications	4
48.	Separate room/space for Pediatric ward/IMNCI clinic	10
49.	Complete episiotomy set	13
50.	Instrumental vaginal delivery sets (vacuum extractor and forceps delivery)	12
51.	Uterine evacuation equipment	12
52.	Complete manual vacuum aspiration set	12
53.	Have a Baby weighing scale	14
54.	Have Cord ties/clips	14
55.	Have Caps or hats to prevent heat loss	6
56.	Have Incubator	8
57.	Have Cup and spoon for infant feeding	9
58.	Have Laryngoscope newborn size	4
59.	Have a Respirator for neonates	7
60.	Equipment for resuscitation within the delivery unit is always accessible	13
61.	Waiting area for maternity patients	12
62.	Functional T.V. in the waiting area	3
	**Standard precautions**	
63.	No patient shared beds before, during, or after obtaining the service in the last 3 months	11
64.	No obstetric or gynecologic patient slept on the floor in the last 3 months	9
65.	No patient delivered on the floor, corridor, or bathroom in the last 3 months	11
66.	Cloths or towels for drying the baby	6

**Fig. 2:**
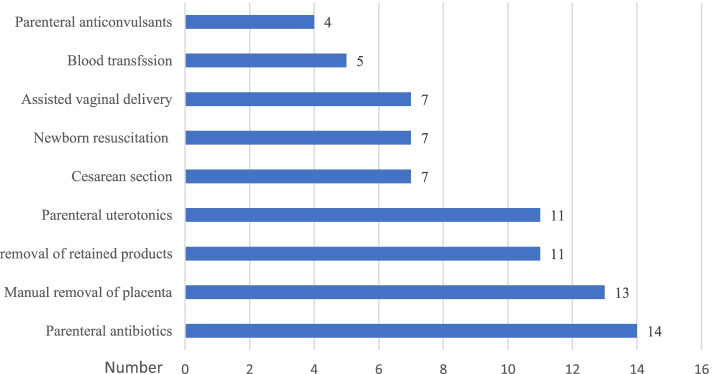
Availability of signal function tests of EmONC services in Wolaita Zone, southern Ethiopia (*n* = 14)

The facility audit showed that 14 (100%) and 13 (92.9%) facilities had given parenteral anti-biotics and manual removal of placenta for the EmONC patients, respectively in the past 3 months. In contrast, only four facilities provided parenteral anticonvulsants to women with EmONC emergencies in the previous 3 months of the study (Fig. 2).

### Observed/process and output quality

**Fig. 3:**
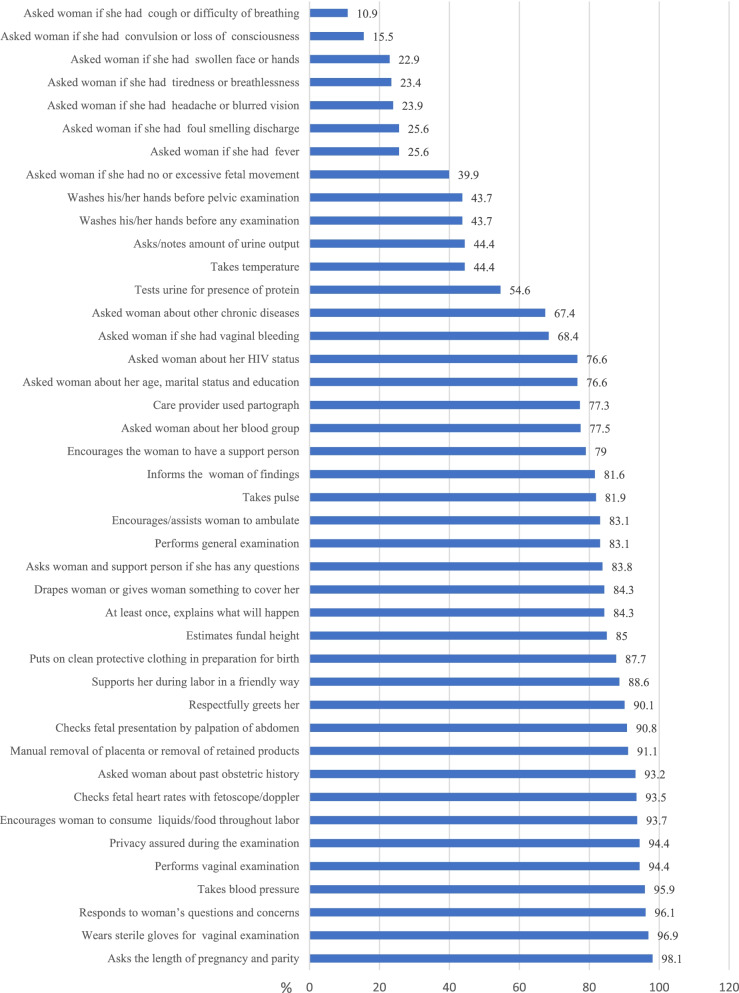
Observed/process quality of EmONC service in Wolaita Zone, southern Ethiopia (*n* = 414)

The mean observed quality index of EmONC services was 69.4% (95% C.I: 67.9, 70.8%) with an SD of 15.4%. The care providers checked clients’ cards or asked the age, gestational age, or parity of 406 (98.1%) of the women receiving the service. Of 414 observations, in 181 (43.7%) and 401 (96.9%) observations, the care providers washed their hands and wore gloves before conducting pelvic examinations respectively. However, only in 10.9 and 15.6% of observations did care providers ask the women whether they had the danger signs of pregnancy such as cough or difficulty breathing and convulsion or loss of consciousness, respectively (Fig. 3). The mean output quality of EmONC service (women’s satisfaction with the service they received) was 79.6% (95% C.I: 78.5, 80.7%) with a standard deviation of 12.1%. Of the 414 women, 245 (59.2%) received below 75% of the standard clinical actions of EmONC service. Only a quarter of women received 81% or more of the standard clinical actions of EmONC service.

### Predictors of observed EmONC services quality

The multiple linear regression analysis (Table 4) showed that women’s education, age, duration of stay at the facility, number of patients in the delivery room, and care provider experience were statistically significant predictors of the observed EmONC service quality. Accordingly, compared to the non-educated women, the average value of the quality index is higher among women with educational status of grades 1–8 by nearly 5% (B = 5.35, 95% C.I: 0.56, 10.14), and grades 9–12 by almost 8% (B = 8.38, 95% C.I: 2.92, 13.85). Similarly, the average quality index was higher among older women (30 years and above) than their younger counterparts by nearly 4% (B = 3.86, 95% C.I: 0.39, 7.33).

**Table 4:** Multiple linear regression on predictors of observed quality of EmONC services in Wolaita Zone, southern Ethiopia (*n* = 414)

Variable	Unadjusted coefficient		Adjusted coefficient			
	B	*P*-value	B	*P*-value	95% C. I for B	
					Lower	Upper
Wealth index (comparator: lower)						
Middle	−4.69	0.03	−3.15	0.10	−6.95	0.65
Higher	−2.16	0.32	−0.25	0.92	−4.90	4.40
Care provider’s sex (comparator: Male)						
Female	1.06	0.49	−2.24	0.17	−5.46	0.98
Occupation of the patient (comparator: Farmer)						
Employed	−0.65	0.77	2.84	0.32	−2.72	8.39
Student	−3.45	0.11	−0.19	0.94	−4.93	4.55
Other^a^	−4.85	0.07	−1.71	0.53	−7.03	3.61
Educational status of patient (comparator: Uneducated)						
Grade 1–8	6.95	0.00	5.35	0.029^*^	0.56	10.14
Grade 9–12	11.71	0.00	8.38	0.003^*^	2.92	13.85
College or university	7.56	0.00	1.46	0.69	−5.80	8.72
Age of patient (comparator: < 30)						
> 30	4.79	0.00	3.86	0.029^*^	0.39	7.33
Duration of stay at the health facility	3.61	0.00	3.58	0.001^**^	2.26	4.90
Number of patients in the delivery room	−5.59	0.00	−4.14	0.001^**^	−6.14	−2.13
Experience of Care provider	1.38	0.00	1.26	0.001^**^	0.83	1.69

* *P*-value < 0.05; ** *P*-value < 0.001; ^a^ daily laborer, housewife, and merchant

For every one-day increase in facility stay, the quality index increased by nearly 4% (B = 3.58, 95% C.I: 2.66, 4.9). However, the number of patients (women receiving EmONC service) in the delivery room was inversely associated with the quality of care provided. Accordingly, for one more woman receiving EmONC service in the delivery room, the average quality of care index decreased by 4% (B = − 4.14, 95% C.I: − 6.14, − 2.13). The care provider’s experience was positively associated with the quality of care, i.e., for every year increase in the care provider’s experience, the quality of care increased by 1.3% (B = 1.26, 95% C.I: 0.83, 1.69).

## Discussion

Enhancing health service quality is the centre for improving maternal and child health and wellbeing [38], which further leads to universal health coverage [39]. Findings from the current study identified that the mean input, process/observed, and output quality for EmONC services in Wolaita Zone, southern Ethiopia, were 74.2, 69.4, and 79.6%, respectively, with a significant variation from facility to facility.

Among the Donabedian’s quality measurements indices, the observed/process quality was the lowest quality of care identified. This finding is in line with a study conducted in eight low- and middle-income countries (LMICs) [40] and northern Ethiopia [18], in which the observed clinical quality was lower than infrastructure. This indicates that the availability of equipment, materials, drugs, and reagents is relatively better than the observed care women received.

Some studies have measured the quality of EmONC services. However, their measurement frameworks and study settings varied considerably [20, 23, 24, 41]. In Tigray (northern Ethiopia), the health facilities provided poor basic EmONC services quality (66.7%) [20], making it similar to this study, although the study had followed a different approach to measuring quality.

A study from the Democratic Republic of the Congo reported that none of the studied health facilities provided high-quality EmOC services [23]. This study focused on measuring the quality of care through five elements: training for staff; availability of guidelines; materials and equipment; and products; and diagnostic capability, such as blood transfusion, [23] mainly focusing on the input component of quality care. Another study from Northern Nigeria [24] reported a worsening trend. In contrast, a study from Mozambique [41] reported improved emergency obstetric care service quality measured through a direct case fatality rate indicator. Although maternal death rate and other similar indices are often used to measure the quality of care in developed countries, they are rarely used in developing countries [25]. This is because they introduce selection bias (since cases with severe and life-threatening outcomes are referred to higher-level health facilities). Hence, the absence of maternal mortality might not necessarily indicate better quality [25, 42]. Nevertheless, evidence emphasized that the observed progress should not be considered a magnificent achievement. Hence, efforts are needed to support the health system to improve the quality of care in obstetric emergencies [41].

Every three in five women in the current study received below 75% of the standard clinical actions of EmONC services. This finding aligns with a study from northern Ethiopia (Tigray region) in which 69.8% of women in that region received poor quality intrapartum care [19]. The resemblance might be due to the similarity in the governing health system, low health professionals’ skills and attitudes in both studies, and comparably poor health infrastructure. Another study conducted in five African countries (Kenya, Namibia, Rwanda, Tanzania, and Uganda) reported that 40% of women received essential maternal care functions from health facilities with poor quality (measured using structure and process of care) [25]. A national survey from Ethiopia also reported that most (86%) women received less than half of the recommended clinical actions [43]. Though the reported difference is attributed to the scope of the studies, study settings, and the components of quality measurement indices, the findings emphasize that women are receiving poor-quality services.

Despite the importance of signal function tests in preventing and treating severe and life-threatening obstetric emergencies, some facilities in the study performed poorly or did not provide any services at all. Accordingly, parenteral antibiotics were the most commonly offered signal function test, whereas parenteral anticonvulsants and blood transfusion were the least provided signal function tests. This finding was in line with a study from the Democratic Republic of Congo in which parenteral antibiotics were among the most delivered signal function tests. In contrast, parenteral anticonvulsant was the least common signal function test [44]. A similar finding was also reported from a study conducted in Nigeria [24] and Mozambique [41]. The similarity of poor performance in signal function tests might be attributable to a shortage of resources in sub-Saharan African countries. Evidence reported that the majority (75%) of maternal death is caused by hemorrhage, infections, hypertensive disorders of pregnancy, obstructed labor, and unsafe abortion [3, 5], which could be prevented and treated through access to functional EmONC facilities. However, the lack of signal function tests in the current study and other similar settings indicate that the health system needs to emphasize the readiness of EmONC facilities’ signal function tests.

The multiple linear regression analysis showed the independent predictors of the observed quality of EmONC services. Accordingly, the patient’s age and educational status were the statistically significant socio-demographic predictors of the quality of EmONC services. Though evidence reported that younger women have a higher risk of obstetric complications and death [3], the current study reported that the average quality of care index increases as the age of women increases. This indicates that the service provision, care providers’ attitude, and the patients’ needs should be well addressed in accordance with the age of mothers, especially the youths. Though the country (Ethiopia) has a youth health strategy for addressing youth’s reproductive and sexual health needs [45], adherence to the guidelines (regarding the quality of EmONC services) appears to be poor.

The patient flow and crowding of health facilities also determine the quality of care in our study. I.e., the higher the number of patients (in the same room), the lower the average quality of EmONC services, keeping other variables constant. This could be because higher patient flow (within the limited health system’s capacity) can further overburden care providers’ work overload and limit the provision of quality emergency services [46]. Besides, evidence supports that crowding in emergency departments hampers quality service through care providers’ inability to adhere to guidelines and adverse treatment outcomes [46]. Our study also identified that though all the studied facilities had a separate room for child delivery, some facilities had a shortage of quality-of-care-related infrastructures, such as separate rooms for labor, maternity, and sufficient beds for patients. Hence, quality without infrastructure is inconceivable, so an input/infrastructure enables the health system to provide quality health service [47].

Furthermore, our study identified that a longer duration of health facility stay was positively associated with better quality service. This might be because of giving attention to severe complications (which need prolonged care) and underestimating management of some complications as less relevant. This could further result in noncompliance with the standards of care and procedures. Evidence also indicated that early and unindicated discharge had a higher risk of dying among emergency patients [48]. Though reducing the obstetric women’s duration of hospital stay helps retain healthcare costs, sufficient beds, and staff contingency, it should be assured that access to quality of care is not compromised [49]. Besides, evidence also reported that earlier discharge results in a significant number of maternal and newborn healthcare needs at their home [50]. Nevertheless, evidence on the extent of hospital stay and its effect on the quality of EmONC service and the health status of those discharged early is still inconclusive, and little is known [51–54].

Finally, this study reported that care providers’ experience was also an independent predictor of the quality of care provided. Accordingly, as the work experience of the care provider increases, the average observed quality of care increases and vice versa. This implies that the incompetence of skilled staff is one of the major causes of poor-quality service provision, which ultimately results in adverse health outcomes, including the *‘third delay*’ (maternal death in health facilities) [46]. Though studies focusing on the association between care providers’ experience and quality of care are limited, evidence indicated that a more diverse staff and skill mix had a positive effect on service quality [55]. However, the current study didn’t address the care providers’ knowledge and skill in managing EmONC complications so that further investigations could support the identified (existing) evidence.

### Strength and limitations of the study

This study is the first to examine the quality of EmONC services using all the three domains of Donabedian’s model (structure, process, and output quality) in Ethiopia. Multiple data collection techniques (facility audit, observation, and exit interviews) enriched the study to yield concrete findings on the quality of EmONC services. Nevertheless, the observational data collection can be suspected of its Hawthorne effect (the reactivity of care providers in response to their awareness of being observed). These phenomena usually last for a few observations (short-lived impact) so we rejected the first five observations to control the effect. Though the cesarean section is one of the components of CEmONC services, women with major obstetric/gynecological surgical procedures (cesarean section, hysterectomy, colporrhaphy, cervical cerclage, etc.) were excluded from the study because such patients need a prolonged hospital stay and need senior (specialist) doctor’s treatment/management. Hence, observation of the content of care for such patients would not be practical.

## Conclusion

The EmONC services quality (measured using input, process (observed), and output quality measures) in Wolaita Zone was sub-optimal. Every three in five women in the current study received less than three-fourths of the standard clinical actions of EmONC services. Though every EmONC facility is expected to provide the signal function tests to prevent and treat severe and life-threatening obstetric emergencies, some facilities were performing poorly or not providing some services at all. Accordingly, parenteral antibiotics were the most commonly given signal function test, whereas parenteral anticonvulsants and blood transfusion were the least provided signal function tests. Finally, the study identified the patient’s age, educational status, duration of stay at the facility, number of patients in the delivery room, and care provider experience as independent predictors of observed quality EmONC service.

### Recommendation

The local/national health system might benefit from emphasizing the availability of equipment, drugs, and other medical infrastructure to improve the quality of EmONC services. The health professionals’ adherence to the standard procedures and guidelines should be improved through training, supervision, and frequent monitoring and evaluation. Enhancing the human resource for health facilities with more experienced care providers could improve the quality of EmONC service. Regardless of the women’s characteristics and medical emergencies, standard care should be provided to every woman who needs the service. Further study should be conducted to identify the care providers’ skills and knowledge, community, and health system factors that affect the utilization of quality EmONC services. The predictors of women’s satisfaction should also be investigated.

## Data Availability

Due to the presence of identifying sensitive information, data can be made available upon reasonable request to the corresponding author.

## Abbreviations

AMDD: Averting Maternal Death and Disability
BEmONC: Basic Emergency Obstetric and Newborn Care
BREC: Biomedical Research Ethics Committee
C.I: Confidence Interval
CEmONC: Comprehensive Emergency Obstetric and Newborn Care
EmONC: Emergency Obstetric and Newborn Care
IESO: Integrated Emergency Surgical Officers
IRB: Institutional Review Board
IV: Intravenous Fluid
LMICs: Low and Middle-Income Countries
MMR: Maternal Mortality Ratio
ODK: Open Data Kit
SD: Standard Deviation
SVD: Spontaneous Vaginal Delivery
UNICEF: United Nations Children’s Fund
UNFPA: United Nations Population Fund
USAID: United States Agency for International Development
VIF: Variance Inflation Factor
WHO: World Health Organization
